# Predictors of PFOA Levels in a Community Surrounding a Chemical Plant

**DOI:** 10.1289/ehp.0800294

**Published:** 2009-03-23

**Authors:** Kyle Steenland, Chuangfang Jin, Jessica MacNeil, Cathy Lally, Alan Ducatman, Veronica Vieira, Tony Fletcher

**Affiliations:** 1 Rollins School of Public Health, Emory University, Atlanta, Georgia, USA; 2 Department Community Medicine, West Virginia University School of Medicine, Morgantown, West Virginia, USA; 3 Department of Environmental Health, Boston School of Public Health, Boston University, Boston, Massachusetts, USA; 4 London School of Hygiene and Tropical Medicine, London, United Kingdom

**Keywords:** PFOA, serum levels, water contamination

## Abstract

**Background:**

Perfluorooctanoic acid (PFOA) is considered a probable human carcinogen by the U.S. Environmental Protection Agency. It does not exist in nature but has been used widely since World War II. It is present in the serum of most Americans at about 4–5 ng/mL, although the routes of exposure remain unknown.

**Objectives:**

We examined predictors of PFOA in mid-Ohio Valley residents living near a chemical plant that until recently released large quantities of PFOA into the environment, contaminating drinking water.

**Methods:**

We studied 69,030 residents in six contaminated water districts who participated in a 2005–2006 survey involving a questionnaire and blood tests. Of these, 64,251 had complete data on PFOA and covariates. We also analyzed a subset (71%) for whom we had occupational history. We ran linear regression models to determine serum PFOA predictors.

**Results:**

Mean PFOA serum level was 83.0 ng/mL (median, 28.2). The most important predictors were current (median for all districts, 38.4; highest district, 224.1) and past (median, 18.6) residence in contaminated water districts, and current (median, 147.8) and past (median, 74.9) employment at the chemical plant (*R*^2^ model = 0.55). PFOA was higher for males, those consuming local vegetables, and those using well water rather than public water, and lower for those using bottled water. PFOA was higher at younger and older ages.

**Conclusions:**

PFOA levels in this population varied with distance of residence from the plant and employment at the plant. Effects of age and sex reflected prior findings. Effects of other demographic and lifestyle covariates were relatively weak.

Perfluorooctanoic acid (PFOA, or C8) does not occur in nature. It is used as a polymerization aid in the manufacture of several types of fluoropolymers, which are used in a wide variety of industrial and consumer products, including extensive use in the manufacture of Teflon. PFOA does not break down once in the environment, leading to widespread buildup and bioaccumulation. The half-life of PFOA in human serum has been estimated to be about 4 years (Olsen et al 2007). Most people in the United States have measurable PFOA in their serum, with a median of 4 ng/mL in 2003–2004 (Calafat et al. 2007b), although the exact sources of this exposure are not clear.

PFOA causes cancer of the testicles, liver, and pancreas in rodents, and there is some evidence that it also causes breast cancer in rodents [U.S. Environmental Protection Agency (EPA) 2005]. It also causes fetal loss and low birth weight in mice and immunotoxic and hepatoxic effects in rodents (U.S. EPA 2005). Health effects in humans are not well established. There have been some reports of associations with lower birth weight (Apelberg et al. 2007; Fei et al. 2007), higher cholesterol (Sakr et al. 2007a, 2007b), and impaired liver function (Olsen et al. 2007), but these effects are usually modest, and the literature is sparse. Mortality studies of workers have shown increases in some causes of death but have not been consistent and have been based on relatively small numbers of deaths (Gilliland and Mandel 1993; Leonard et al. 2007).

PFOA has been used in the manufacturing of fluoropolymers at a chemical plant in Washington, West Virginia, since 1951, with use peaking in the late 1990s. PFOA is used a surfactant in the polymerization of trifluoroethylene to make Teflon. It entered the groundwater via both air emissions, which were deposited on the soil around the plant and leached downward, and emissions into the Ohio River, which then entered the groundwater that communicates with the river. Public drinking water comes from wells pumping from the groundwater, which are located close to the river. Some local landfill sites may have also contributed to ground-water contamination. Emissions have been sharply reduced in the past few years. There is evidence that drinking water is the primary route of exposure for the population living in these districts (Emmett et al. 2006).

In 2001, a group of residents from the Ohio and West Virginia communities in the vicinity of the plant filed a class-action lawsuit alleging health damage due to contamination of human drinking water supplies with PFOA. The settlement of the class action lawsuit led to a baseline survey, called the C8 Health Project, which was conducted in 2005–2006 and gathered data from 69,000 Ohio and West Virginia residents who lived in six contaminated water districts surrounding the chemical plant. The C8 Health Project included blood draws and subsequent measurement of serum PFOA (West Virginia University 2009). The present study is an analysis of these data to study factors associated with PFOA levels.

## Materials and Methods

### Study participants

**Figure 1:**
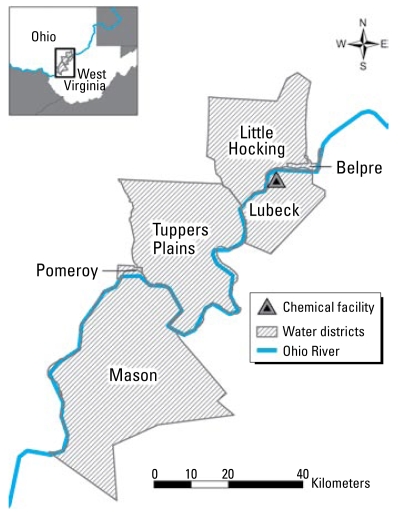
Six contaminated water districts of the C8 Health Project.

The C8 Health Project, conducted by Brookmar Inc., began data collection in August 2005 and completed it in August 2006. Its purpose was to collect health data from class members through questionnaires and a battery of blood tests, including a test to ascertain the concentration of PFOA in the serum. Subjects were eligible to participate in the C8 Health Project if they had consumed drinking water for at least 1 year before 3 December 2004 supplied by Little Hocking Water Association (Ohio), City of Belpre (Ohio), Tuppers Plains Chester Water District (Ohio), Village of Pomeroy (Ohio), Lubeck Public Service District (West Virginia), Mason County Public Service District (West Virginia), or private water sources within these areas that were contaminated with PFOA. Subjects were also eligible if they could document that they had either worked in a contaminated water district or went to school there for at least 1 year. Figure 1 shows the six water districts. Subjects were compensated $400 if they filled out the extensive questionnaire and came to local survey stations to donate a blood sample. A full description of the C8 Health Project is in preparation.

The C8 Health Project collected data on 69,030 subjects. It is not known what percentage of the eligible population participated, because the eligible population was not enumerated (the past populations of the water districts are not known, nor are the number of eligible people who lived outside the water districts but went to school or worked there). Nonetheless, it is believed that most participated, given the widespread public interest and the financial incentive. We have estimated the participation rate among current residents in 2005–2006 among adults ≥ 20 years of age using census data (the population ≥ 18 years of age, as studied here, was not available from the census). Estimates of the population of the six water districts were made based on population estimates for census block groups in 2005. Block groups are smaller than census tracts but larger than census blocks. To find the population of each water district, we determined which block groups were entirely within the water district. We then determined which block groups intersected the boundaries of the water districts. For those which intersected, we then calculated the ratio of water district area to block group area within each block group and multiplied the ratio by the block group population. We then summed the populations for the entire water district and then summed across all six water districts. Finally, we determined the numbers of current residents (63% of total participants) in the water districts who participated in the C8 Health Project in 2005–2006, and divided this number (33,001 residents) by the population (40,721 residents) to find an estimated participation rate of 81% among current residents ≥ 20 years of age.

### Statistical analysis

It was expected *a priori* that water districts would play an important role in predicting exposure, with subjects in water districts more distant from the plant likely to have lower serum levels. Subjects in the C8 Health Project were required to document past or present consumption of contaminated public water from one of the six contaminated water districts (either via living in the water district for at least 1 year, or by working or going to school there for at least 1 year; *n* = 68,873), or having drunk from private wells with documented contamination (*n* = 157). This documented water district of exposure is called the “qualifying” water district. Sixty-three percent of the population reported currently drinking public water (as their main water source) in one of the six water districts. We classified water district into 12 groups: six for currently (2005–2006) drinking public water in one of the six contaminated water districts, and six for not currently drinking public water but having previously been exposed by drinking water in one of the six water districts. Among those classified by their qualifying water district, 73% of these had a record of having lived or worked in the past in their qualifying water district, with the remainder presumably having gone to school there (no data were available on school history). Hereafter, we loosely describe these variables as “current” (2005–2006) and “past” exposure, because most of those not currently drinking contaminated public water qualified for the study because of having drunk contaminated water in the past.

Besides age, race, sex, and water district, other *a priori* variables of interest were having worked at the chemical plant, growing your own vegetables, and drinking bottled water (Emmett et al. 2006). Detailed employment history was available for only adult study subjects who consented to make available identifiable information to the authors as part of future follow-up studies (71%). We restricted analyses using a variable for current or past employment at the chemical plant to that subset. Our initial model was based on including these *a priori* variables found to be important in previous studies, as well as the variables for water district. The initial model then included current or past water district, occupational exposure (for the subset with available data), eating local vegetables, use of bottled water, age, sex, and race (white vs. nonwhite).

We included the entire population (*n* = 69,030) in analyses using the above regression model (absent occupational exposure, available on a subset), to which we added a large number of other potentially important variables, ultimately retaining those that had a significant association (at *p* ≤ 0.05) with PFOA. Because the population is so large and any variable only slightly associated with PFOA may be statistically significant, this strategy of model building led to inclusion in the final model of variables statistically associated with PFOA levels but without any important contribution to explaining the overall variance of PFOA. We adopted this strategy partly because of the exploratory nature of this analysis and the minimal prior data on factors associated with PFOA in the general population. Results from regression models in which the log of PFOA was the outcome were transformed back to the original unlogged scale, resulting in multiplicative effects for predictor variables. All predictor variables in the regression were categorical. Predicted values were reported as a percent change compared with baseline values for each categorical variable in the regression.

All regression models used the natural log transformation of PFOA because the log transform was more normally distributed; we checked residuals for normality.

### Laboratory method for PFOA

Analyses were conducted by a large commercial lab (Exygen, State College, PA, USA). PFOA is customarily measured in the serum, where virtually all PFOA in whole blood may be found (Ehresman et al. 2007). The analytical method for measurement of PFOA in the serum, which was used in this study, has been described in detail previously (Flaherty et al. 2005; Longnecker et al. 2008). Briefly, the method uses liquid chromatography separation with detection by tandem mass spectrometry. The approach allows for rapid throughput using a 96-well plate and can handle large numbers of samples. Extraction of the serum or plasma samples was done using acetonitrile. Chromatography on the extract was done using a quaternary pump and vacuum degasser. The mobile phases consisted of two systems: a 2 mM ammonium acetate solution, and methanol with gradients set up to ensure both rapid and complete separation. The lab used ^13^C-PFOA at a concentration of 1 ng/mL as its internal standard. Mass spectrometry was done in selected reaction monitoring mode with *m*/*z* = 413 → 369 as the principal ion monitored for PFOA (*m*/*z* = 370 for the ^13^C internal standard). Fortification recoveries using rabbit serum or plasma as the matrix for PFOA were generally within 90–110%. The coefficient of variation based on multiple samples between batches was generally ≤ 0.10 over the range of 0.5–40 ng/mL, with a more precise relative coefficient of variation of approximately 0.01 for highly fortified (10,000 ng/mL) samples (Flaherty et al. 2005).

The limit of detection for PFOA was 0.5 ng/mL. Only 0.06% of observations were below the limit of detection, and we assigned these a value of 0.25 ng/mL.

## Results

**Figure 2:**
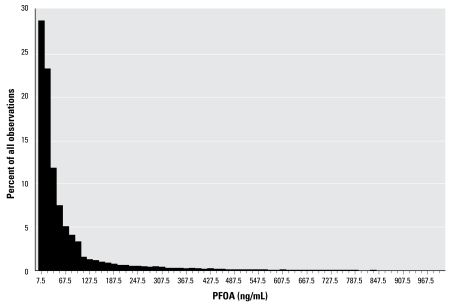
Distribution of PFOA (C8; 405 observations > 1,000 ng/mL not shown).

**Figure 3:**
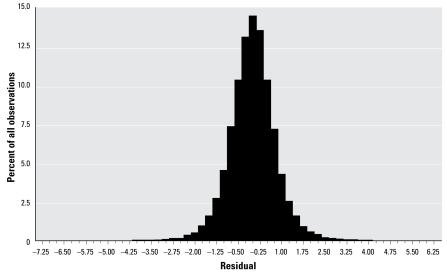
Distribution of residuals from regression model (Table 2).

Table 1 provides descriptive data for the population. Figure 2 shows the distribution of PFOA. The log of PFOA is more normally distributed than PFOA, and use of it in the regression model for the full population led to residuals that were approximately normally distributed (Figure 3). The theoretical 2.5% tails of the distribution of the studentized residuals (> 1.96 or < −1.96) contained 2.76% and 2.60% of the data, respectively, conforming reasonably to expectations.

Table 2 shows the results of the final model for the entire population, without inclusion of a variable for working at the chemical plant (model *R*^2^, 0.55). We added five additional variables [date of testing divided into bimonthly intervals, alcohol consumption in the last 3 days, being a vegetarian, body mass index (BMI), and regular exercise] to variables of *a priori* interest in the initial model, based on each being significantly associated with PFOA (*p* < 0.05) when added to the initial model.

**Figure 4:**
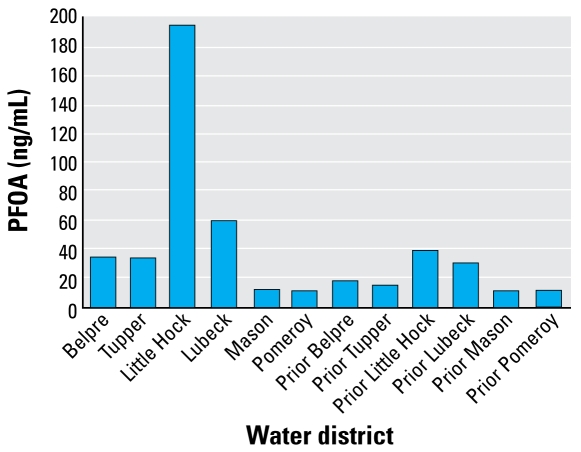
PFOA level (geometric mean) by current and former water district. Current water district refers to living in exposed water district in 2005–2006 at time of blood draw. Prior water district refers to having either lived, worked, or gone to school for at least 1 year in one of the six exposed water districts. Model prediction compared with observed median value of 11.50 ng/mL for Prior Pomeroy.

Table 2 shows strong effects of water district, with current residence in water districts closest to the plant having the highest PFOA levels. Figure 4 shows the data graphically. Currently drinking public water in Little Hocking or Lubeck is associated with the highest levels of PFOA. The well field for Little Hocking public water is located directly across the river from the plant, and the plant itself is located in Lubeck. Current residence in Belpre and Tupper Plains water districts had the next highest levels. These districts are slightly farther away (Belpre is also upstream). Residents of districts farthest away (Mason, Pomeroy) had the lowest levels. Past consumption of water in Little Hocking or Lubeck was also associated with elevated levels, although less than for those currently residing in these water districts. The median level for current residents of any water district was 38.4 ng/mL, whereas the median for past residents was 18.6 ng/mL.

**Figure 5:**
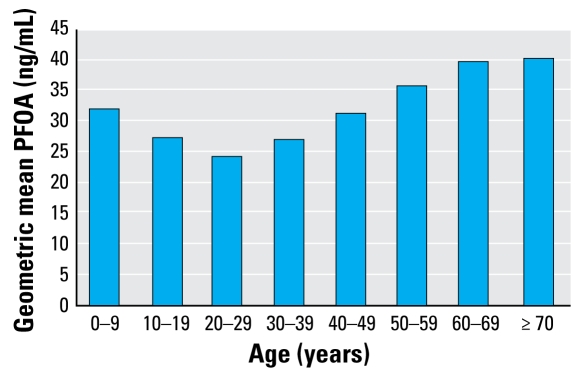
Predicted PFOA serum level (geometric mean) by age: model prediction compared with observed median value of 32.0 ng/mL for age group 0–9 years.

Figure 5 indicates that PFOA levels listed in Table 2 show a J-shaped curve with age. Male sex was also strongly associated with increased PFOA levels. Variables other than water district, age, and sex explained less of the variation in PFOA level.

Growing one’s own vegetables was associated with increased PFOA, whereas drinking bottled water was associated with decreased PFOA. Drinking well water, current smoking, and drinking alcohol in the last 3 days were positively associated with an increase in PFOA. The alcohol finding could reflect some unknown aspect of increased liver activity (protein and lipid production).

Table 2 shows an approximate 30% decrease in levels over the year of testing (2005–2006), which results largely from residents outside the six water districts (37%) who were no longer exposed and whose blood levels dropped as they excreted PFOA. Note that the 34% decrease over time contrasts with the much sharper decrease seen in the unadjusted data in Table 1; this greater decrease reflects the greater participation of residents from low-exposure areas toward the end of the year-long study, which does not appear in the adjusted results in Table 2, based on the model in which we included water district as a variable.

High BMI was associated with lower PFOA levels. White race slightly increased PFOA but was not statistically significant. We did not include socioeconomic status (SES) in the model. There was a weak positive trend between household income and PFOA concentration, and a stronger (contradictory) negative trend between years of schooling and PFOA for those ≥ 30 years of age. These conflicting results do not lend themselves to any simple conclusion regarding an association of PFOA and SES.

We conducted further analyses restricting the data set to the 71% of the population with employment history, and adding a variable for working at the chemical plant to the model in Table 2. Overall, the *R*^2^ for the model for this subset was 58%, similar to the *R*^2^ of 55% for the model with all subjects. Currently working at the plant was associated with a much higher level of PFOA [coefficient = 1.41; standard error (SE) = 0.03; *p* < 0.0001, partial *R*^2^ = 0.06], equivalent to a 309% increase in PFOA compared with someone who had never worked at the plant. Prior work at the plant was also associated with a higher level (coefficient = 0.44; SE = 0.02; *p* < 0.0001; partial *R*^2^ = 1%), equivalent to a 55% increase in PFOA compared with someone who never worked at the plant. Coefficients for other variables remained largely unchanged, with the exception of the coefficient for white versus nonwhite, which increased from 0.020 to 0.064 (SE = 0.023; *p* = 0.005). Working at the chemical plant was slightly less common for whites than for nonwhites [odds ratio adjusted for age = 0.79; 95% confidence interval (CI), 0.62–1.00], such that inclusion of a variable for working at the plant may have made the estimate of race more accurate (i.e., occupational exposure was a negative confounder for the effect of white vs. nonwhite).

As a sensitivity analysis, we reran the model in Table 2 after eliminating the top 1% and bottom 1% of the distribution of studentized residuals, to consider the possible influence of outliers. This analysis, with 98% of the original data, increased the *R*^2^ of the model from 55% to 63%, as might be expected. However, this led to little change in most model coefficients, especially the most important predictors. All the same variables were statistically significant or not statistically significant as in the original analysis, with the exception of race, which became statistically significant without the outliers. The coefficients for age, sex, current water district, BMI (> 30), prior water district, date of testing, growing your own vegetables, being a vegetarian, current alcohol consumption, and using well water changed by ≤ 10%. The coefficients for race, current and former smoking, BMI (first two categories), and using bottled water changed by > 10%, indicating they were more affected by outliers. They were among the least important predictors, none of which had a partial correlation coefficient > 1%; the coefficients for race, BMI (first two categories), and former smoking were not statistically significant in the full model.

Similarly, for sensitivity analysis we restricted the analysis to 50% of the data after generating a uniform random number and taking those in the lower half, to see how stable our results were. The model *R*^2^ was again 55%. In this analysis, however, there was more variation in the estimated model coefficients. Ten of 35 coefficients changed > 20%, although all had the same sign (positive or negative). Those that changed were among the least stable; 7 of the 10 were not significant at the 0.05 level in the split sample, and six of these had not been significant in the original complete data analysis. Overall, all coefficients had the same direction (i.e., were consistently positive or negative in the full and 50% split sample).

## Discussion

PFOA is an important chemical introduced after WWII and now found in virtually the entire U.S. population. The routes of exposure in the general population are not known. PFOA is known to have some toxic properties in animals, but no human health effects have been clearly established.

Data remain sparse on factors associated with serum levels of PFOA. Two prior studies of the general population [National Health and Nutrition Examination Survey (NHANES) population, 1999–2000, and NHANES 2003–2004] found that males had higher levels, that there was little trend with age, that whites had higher levels than Hispanics and blacks, and that increased education was associated with higher serum levels of PFOA (Calafat et al. 2007a, 2007b). *R*^2^ values for regression models were not reported. These findings sometimes failed to reach statistical significance and sometimes were apparent only in certain age groups. Both studies were restricted to adults. Further research using NHANES data for children has shown that children had higher PFOA levels than did adults (Calafat A, personal communication, May 2008). PFOA levels in the United States may be decreasing in the past several years since several manufacturers have stopped or drastically reduced the use of PFOA (Calafat et al. 2007b; Olsen et al. 2007).

Olsen et al. (2007) studied 140 Red Cross donors in 2000 and 2005 with background levels of exposure and found that men had significantly higher serum levels of PFOA than did women but that there were no trends with age. Kannan et al. (2004) studied 473 serum samples from many countries and found that PFOA was present in most samples from industrialized countries but found no significant differences by sex or age. Emmett et al. (2006) studied 371 highly exposed subjects drinking PFOA-contaminated water (median level ~ 354 ng/mL), residing near the same plant under study here. They found a J-shaped relationship with age (high exposure at young and old ages). They also found that eating locally grown vegetables increased PFOA levels, whereas drinking bottled water decreased serum PFOA levels. Work at the nearby plant sharply increased PFOA levels in serum. Hölzer et al. (2007) studied 355 exposed and 236 nonexposed community subjects in Germany. The exposed subjects drank water contaminated with fluoropolymers, predominantly PFOA; the average PFOA serum level was approximately 25 ng/mL. Factors significantly associated with higher PFOA levels were male sex, higher age, drinking larger quantities of public water, eating local vegetables, and residing in the exposed versus nonexposed area.

Here we have studied such factors in by far the largest population to date. This population has been exposed to PFOA primarily through drinking water contamination from a nearby plant, as did the population of 600 studied in Germany (Hölzer et al. 2008).

We have found that markedly higher levels of PFOA were associated with working at the chemical plant that was the source of the contamination. Workers who no longer worked at the plant had much higher levels (median, 75) than did nonworkers (median, 24) but lower levels than those who continued working there (median, 148), consistent with a gradual excretion of PFOA from the body after ending high exposure. Other occupational data (Sakr et al. 2007a) have shown that 1,000 workers at the plant in 2004 had a mean serum level of 428 ng/mL. This is virtually identical to the mean serum level we have found in our data for the subset of workers currently at the plant (427 ng/mL) in 2005–2006 (the PFOA distribution among workers was highly skewed, accounting for difference between the mean and median serum levels, 427 versus 147 ng/mL for current workers).

The other main factor influencing PFOA levels in the population studied here was the distance of residence from the plant. Current residence in water districts near the plant (e.g., Little Hocking and Lubeck) was associated with the highest levels. Those with prior residence near the plant also had high levels, but much less than those living there currently, again consistent with the gradual excretion of PFOA once high exposure ceases. This analysis via distance of water district from the plant is crude; a more comprehensive analysis using geocoding of past and present addresses, as well as estimates of annual emissions from the plant, is under way.

Demographic and other environmental factors played much less important roles. Male sex was the most important demographic factor associated with higher levels. Age showed a J-shaped relationship with serum PFOA, with higher levels in the young and the old, similar to what has been found previously by Emmett et al. (2006) and Calafat et al. (personal communication, 2008). The reasons for these demographic patterns are not known. We also found a trend of decreasing levels of PFOA over time during this 1-year study, which was primarily due to decreasing levels among people no longer living in the six water districts and therefore no longer exposed.

In conclusion, PFOA levels are far above background in this population that has consumed contaminated drinking water. Further studies are under way to determine whether PFOA is associated with health effects in this population.

## Figures and Tables

**Table 1:** Descriptive statistics of mid-Ohio Valley residents exposed to PFOA (*n* = 69,030).

Variable^a^	No. (%)	Median PFOA (ng/mL)
Blood PFOA in 2005–2006	69,030 (100)	28.2^b^

Age (years)		

0–9	4,915 (7.1)	32.8
10–19	9,658 (14.0)	26.6
20–29	10,073 (14.6)	21.0
30–39	10,547 (15.3)	22.7
40–49	12,113 (17.6)	28.0
50–59	10,515 (15.2)	33.6
60–69	6,881 (10.0)	42.9
≥ 70	4,328 (6.3)	40.1

Sex		

Male	33,242 (48.2)	33.7
Female	35,788 (51.8)	23.7

Race		

White	66,989 (97)	28.1
Nonwhite	2,041 (3)	29.5

BMI		

< 24	18,849 (28.1)	27.9
24–26	12,501 (18.6)	29.1
27–29	11,800 (17.6)	30.8
≥ 30	24,005 (35.8)	26.1

Worked at chemical plant^c^		

Yes, current	1,171 (2.4)	147.8
Yes, previous	1,447 (2.9)	74.9
No	45,276 (94.9)	24.3

Grow own vegetables		

Yes	16,015 (23.2)	34.1
No	53,015 (76.8)	26.7

Currently resident in water district		

Belpre	5,388 (7.8)	35.0
Tupper Plains	9,703 (14.1)	37.2
Little Hocking	8,390 (12.2)	224.1
Lubeck	8,289 (12.0)	66.9
Mason County	10,066 (14.6)	12.4
Pomeroy	1,560 (2.3)	12.1

Previously resided or worked in water district		

Belpre	3,387 (4.9)	17.3
Tupper Plains	4,359 (6.3)	13.6
Little Hocking	4,465 (6.5)	33.7
Lubeck	8,552 (12.4)	28.4
Mason County	2,711 (3.9)	10.5
Pomeroy	2,016 (2.9)	11.0

Vegetarian		

Yes	502 (0.7)	24.5
No	68,528 (99.3)	28.2

Consumed alcohol in last 3 days		

Yes	8,883 (13.1)	33.4
No	59,029 (86.9)	27.6

Current smoking		

Yes	14,847 (21.5)	25.3
No	54,088 (78.5)	29.3

Former smoking		

Yes	14,697 (21.3)	31.2
No	54,280 (78.7)	27.5

Regular exercise		

Yes	22,072 (32.0)	30.3
No	46,958 (68.0)	27.3

Bottled water		

Yes	3,728 (5.4)	31.3
No	65,302 (94.6)	28.0

Well water		

Yes	4,434 (6.4)	21.7
No	64,596 (93.6)	28.7

Date of testing		

First 2 months	10,284 (14.9)	48.9
Second 2 months	14,046 (20.4)	39.9
Third 2 months	15,524 (22.4)	28.8
Fourth 2 months	14,948 (21.7)	23.8
Fifth 2 months	8,756 (12.7)	17.8
Last 2 months	5,472 (7.9)	14.7

a A total of 2,120 subjects were missing PFOA values, 1,875 subjects BMI, 1,118 subjects alcohol use, 95 subjects current smoking, 53 subjects former smoking, 8,649 subjects household income, and 144 subjects water district.

b Mean 83.6 ng/mL, geometric mean 32.9 ng/mL.

c Data on working at chemical plant were available for only 71% of the population.

**Table 2:** Multiple linear regression model for the log of PFOA level in all six water districts (model *R*^2^ = 0.55, *n* = 64,251).

Variable	Predicted change (%) in PFOA vs. referent group	Regression coefficient [change in log PFOA (95% CI)]	*p*-Value	Variance (%) in PFOA (partial *R*^2^)
Age (years)				

0–9	Referent			
10–19	−15	−0.16 (−0.20 to −0.12)	< 0.0001	< 1
20–29	−24	−0.28 (−0.32 to −0.24)	< 0.0001	< 1
30–39	−16	−0.17 (−0.21 to −0.13)	< 0.0001	< 1
40–49	−2	−0.02 (−0.06 to 0.02)	0.24	< 1
50–59	12	0.11 (0.07 to 0.15)	< 0.0001	< 1
60–69	23	0.21 (0.17 to 0.25)	< 0.0001	< 1
≥ 70	26	0.19 (0.11 to 0.27)	< 0.0001	< 1

Sex				

Female	Referent			
Male	35	0.30(0.29 to 0.31)	< 0.0001	2.9

BMI				

< 24	Referent			
24–26	2	0.02 (−0.01 to 0.03)	0.13	< 1
27–29	2	0.02 (−0.01 to 0.03)	0.18	< 1
≥ 30	−4	−0.04 (−0.05 to −0.01)	< 0.0001	< 1

Grow vegetables				

No	Referent			
Yes	11	0.10 (0.08 to 0.12)	< 0.0001	< 1

Currently resident in water district				

Belpre	203	1.11 (1.07 to 1.15)	< 0.0001	3.7
Tupper Plains	200	1.10 (1.06 to 1.14)	< 0.0001	4.1
Little Hocking	1,612	2.84 (2.80 to 2.88)	< 0.0001	21.5
Lubeck	421	1.61 (1.61 to 1.69)	< 0.0001	8.2
Mason County	9	0.09 (0.05 to 0.13)	< 0.0001	< 1
Pomeroy	3	0.03 (−0.03 to 0.09)	0.27	< 1

Previously lived or worked in water district				

Prior Belpre	62	0.48 (0.44 to 0.52)	0.005	< 1
Prior Tupper Plains	36	0.29 (0.25 to 0.33)	< 0.0001	< 1
Prior Little Hocking	246	1.22 (1.18 to 1.26)	< 0.0001	4.3
Prior Lubeck	169	0.88 (0.84 to 0.92)	< 0.0001	3.2
Prior Mason County	−2	−0.01 (−0.05 to 0.04)	0.57	< 1
Prior Pomeroy	Referent			

Vegetarian				

No	Referent			
Yes	−10	−0.10 (−0.18 to −0.02)	0.01	< 1

Consumed alcohol in last 3 days				

No	Referent			
Yes	7	0.06 (0.04 to 0.08)	< 0.001	< 1

Smoking				

Never	Referent			
Current	6	0.06 (0.04 to 0.08)	< 0.0001	< 1
Former	−1	−0.01 (−0.03 to 0.01)	0.18	< 1

Bottled water				

No	Referent			
Yes	−6	−0.06 (−0.08 to −0.04)	< 0.0001	< 1

Well water				

No	Referent			
Yes	12	0.11 (0.09 to 0.13)	< 0.0001	< 1

Race				

Nonwhite	Referent			
White	2	0.02 (−0.02 to 0.06)	0.31	< 1

Time of blood draw				

First 2 months	Referent			
Months 3–4	6	0.06 (0.04 to 0.08)	< 0.0001	< 1
Months 5–6	−11	−0.12 (−0.14 to −0.10)	< 0.0001	< 1
Months 7–8	−14	−0.15 (−0.17 to −0.13)	< 0.0001	< 1
Months 9–10	−22	−0.25 (−0.27 to −0.23)	< 0.0001	< 1
Months 11–12	−29	−0.34 (−0.38 to −0.30)	< 0.0001	< 1
